# Sexual dimorphism in a mouse model of Friedreich’s ataxia with severe cardiomyopathy

**DOI:** 10.1038/s42003-024-06962-4

**Published:** 2024-10-03

**Authors:** Lili Salinas, Claire B. Montgomery, Francisco Figueroa, Phung N. Thai, Nipavan Chiamvimonvat, Gino Cortopassi, Elena N. Dedkova

**Affiliations:** 1 Department of Molecular Biosciences, University of California, Davis, CA USA; 2 Department of Internal Medicine, University of California, Davis, CA USA; 3 Department of Veterans Affairs, Northern California Health Care System, Mather, CA USA; 4 Department of Basic Sciences, California Northstate University, Elk Grove, CA USA

**Keywords:** Heart failure, Heart failure

## Abstract

Friedreich’s ataxia (FA) is an autosomal recessive disorder caused by reduced frataxin (FXN) expression in mitochondria, where the lethal component is cardiomyopathy. Using the conditional *Fxn*^flox/null^::MCK-Cre knock-out (*Fxn*-cKO) mouse model, we discovered significant sex differences in the progression towards heart failure, with *Fxn*-cKO males exhibiting a worse cardiac phenotype, low survival rate, kidney and reproductive organ deficiencies. These differences are likely due to a decline in testosterone in *Fxn*-cKO males. The decrease in testosterone was related to decreased expression of proteins involved in cholesterol transfer into the mitochondria: StAR and TSPO on the outer mitochondrial membrane, and the cholesterol side-chain cleavage enzyme P450scc and ferredoxin on the inner mitochondrial membrane. Expression of excitation-contraction coupling proteins (L-type calcium channel, RyR2, SERCA2, phospholamban and CaMKIIδ) was decreased significantly more in *Fxn*-cKO males. This is the first study that extensively investigates the sexual dimorphism in FA mouse model with cardiac calcium signaling impairment.

## Abstract (web summary)

Sexual dimorphism is identified in a mouse model of Friedreich’s ataxia, with males exhibiting a worse cardiac phenotype, low survival rate, as well as kidney and reproductive organ deficiencies.

## Introduction

Friedreich’s ataxia (FA) is a rare autosomal recessive disorder caused by a guanine-adenine-adenine (GAA) trinucleotide repeat expansion in the first intron of *Fxn* gene encoding protein called frataxin (FXN)^1^. Typically, healthy individuals have 6–30 GAA repeats in *Fxn* gene; however, the number of GAA repeats in FA patients could vary between 44 and 1700, which causes transcriptional pausing and decreased FXN protein expression in mitochondria^2^. FXN is localized in the mitochondrial matrix where it binds iron and helps to assemble clusters of iron and sulfur (Fe-S) molecules that are critical for the function of many mitochondrial enzymes including aconitase, mitochondrial complexes I, II and III^3–5^. Impaired function of these mitochondrial enzymes and complexes leads to decreased energy production, excessive reactive oxygen species (ROS) generation, and a shift in the mitochondrial redox environment. The severity of disease directly correlates with the number of GAA repeats (increased number of GAA repeats in *Fxn* gene translates into more severe and faster progressing disorder) and inversely correlates with the onset of disease^6^. In the vast majority of cases, FA is diagnosed in children between age 5 and 15-years-old^7^. Late diagnosis after 25 years old is also possible. Although rare, FA is the most common form of hereditary ataxia in the United States, affecting about one in every 50,000 people.

Although this is a cerebellar ataxia which causes progressive damage to the nervous system and movement problems, the lethal component of this disorder is development of left ventricular (LV) cardiomyopathy with either preserved or reduced LV ejection fraction (EF)^8–11^. Patients with a severe decrease in EF tend to have a lower life expectancy than those with preserved EF, and age of disease onset generally correlates with reduced life expectancy^6^. The average life expectancy is about 38 years of age in patients who were diagnosed with FA early in their life^12,13^. It is still ill understood what factors contribute to the severity of cardiomyopathy and premature death in FA patients. Clinical data in FA patients are hard to accumulate due to a small number of patients worldwide and their limited ability to travel to the clinical centers with expertise in FA. Moreover, animal models of FA often do not represent all clinical symptoms of disease observed in patients with FA. While there are several mouse models of FA currently available, it has proven difficult to find a model that accurately recapitulates cardiomyopathy observed in individuals with FA^14^. Therefore, mouse models need to be chosen carefully depending on the specific aspect of the disorder being studied.

In this study, we aimed to characterize a recently developed *Fxn*^flox/null^::MCK-Cre mouse model of FA where animals progress rapidly towards severe cardiomyopathy and die prematurely due to FXN loss in the heart. This mouse model is similar to that generated by Dr. Puccio and colleagues^15,16^, but in contrast to Puccio’s model, this mouse model (referred here as the *Fxn*-cKO) contains a Cre conditional knockout of *Fxn* in the presence of muscle creatine kinase (MCK) on a floxed frataxin exon 2 allele. While the floxed exon is different (in Puccio’s model FXN was knocked out on a floxed *Fxn* exon 4 allele), we believe these models function the same phenotypically. Upon studying this mouse model, we discovered significant sex differences between male and female KO mice in both survival and functional changes over their lifetime. Specifically, we found that heart, kidney, and reproductive organs function in the *Fxn*-cKO males was more severely affected by the FXN deficiency as compared to females. The sexual dimorphism has not been previously reported in mouse models of FA. Additionally, this mouse model recapitulates the findings reported in limited clinical studies where male FA patients exhibited significantly higher cardiac hypertrophy, wall thickness, and decreased cardiac function^6,17^. This discovery is critical as this mouse model often serves as a model for therapeutic drug development and testing in FA. Importantly, the study supports clinical findings that FA may be more severe in male individuals. Our study also reveals that in contrast to pressure-overload-induced heart failure (HF) and dilated cardiomyopathy where expression of sodium-calcium exchanger (NCX) and calmodulin-dependent protein kinase II delta (CaMKIIδ) are typically upregulated, cardiomyopathy in FA was associated with decreased expression in NCX and CaMKIIδ in both males and females.

## Results

### *Fxn*-cKO males lose weight and die significantly faster compared to *Fxn*-cKO females

**Fig. 1:**
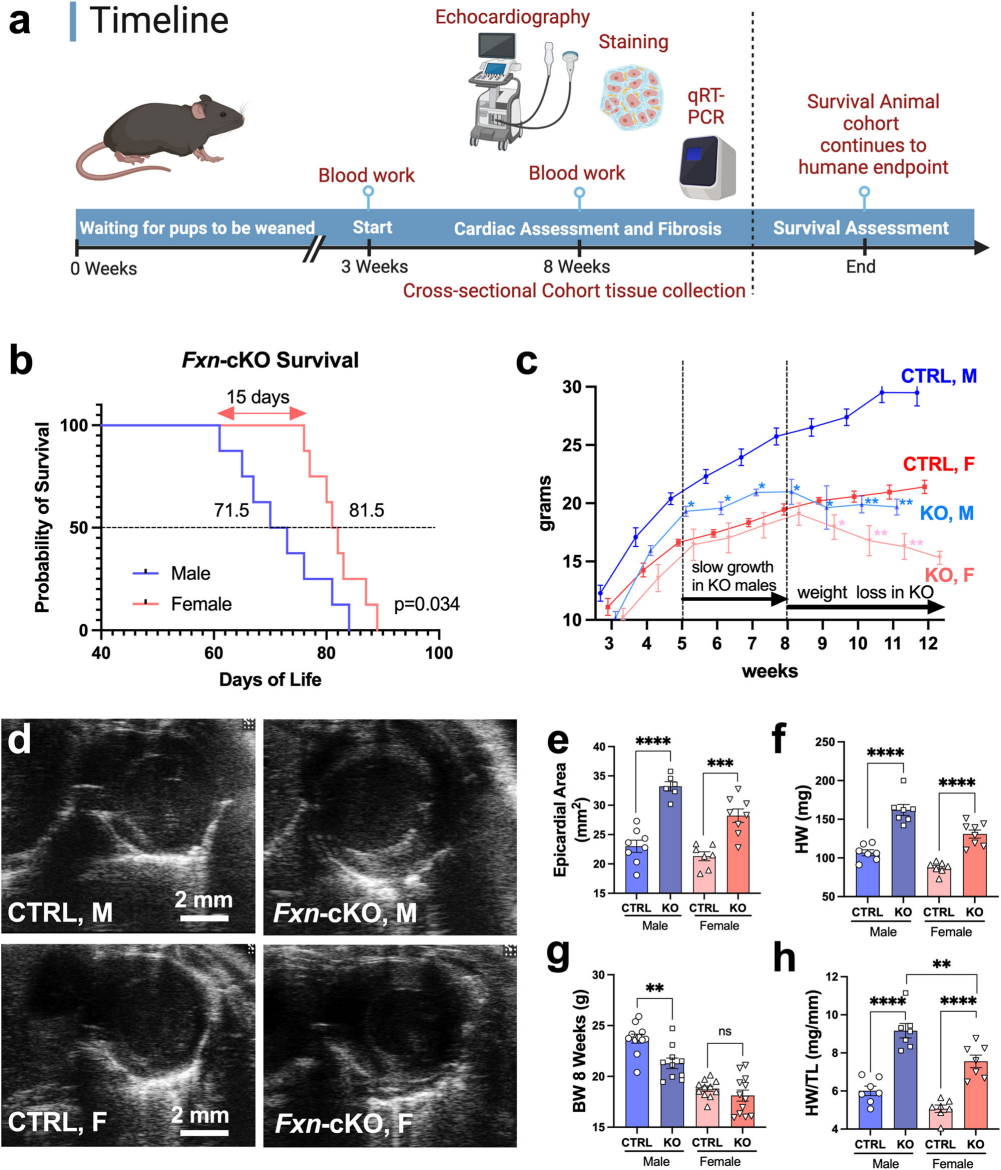
Experimental design, survival and cardiac structure in *Fxn*-cKO mice. **a** Animal experimental design and timeline. Created with BioRender.com. **b** Kaplan-Meier Survival Curve in *Fxn*-cKO mice. Median age of death: males=71.5 (n = 8); females= 81.5 (n = 8) with the Log-rank test p = 0.0367. **c** Body weight changes over lifetime (n = 8 for each group). **d** Representative B mode echocardiography images of CTRL and *Fxn*-cKO hearts. **e** Epicardial area measurements in CTRL vs KO mice. **f** Heart weight measurements in CTRL vs KO mice at 8 weeks of age. **g** Body weight measurements in CTRL vs KO mice at 8 weeks of age**. h** Heart weight normalized to tibia length in CTRL vs KO mice at 8 weeks of age. Significance was determined by two-way ANOVA with *p < 0.05, **p < 0.01, ***p < 0.001, and ****p < 0.0001. NS – non-significant.

The *Fxn*-cKO mouse model used in this study was recently generated in the Jackson lab as a novel mouse model of severe cardiomyopathy in FA. In contrast to previously described FA mouse models^15,16^, the *Fxn*^flox/null^::*MCK-Cre* genotype is compound heterozygous at the frataxin locus (floxed exon 2 and global knockout on respective homologous chromosomes) and hemizygous for MCK-Cre. To characterize this FA mouse model in detail, two experimental cohorts were generated with a timeline reflected in Fig. 1a. In the first experimental cohort, we monitored animals daily until death to examine their lifespan; and in the second cross-sectional cohort of animals, we subjected the mice to functional tests and sacrificed them at 8 weeks of age, prior to any deaths occurring, as determined in the survival study (Fig. 1b). It was previously reported that *Fxn*-cKO animals with the conditional deletion of mouse *Fxn* at exon 4 die prematurely due to severe cardiomyopathy with a median of 78.5 days of survival and overall lifespan of 76 ± 10 days^15,18^. We, however, observed that *Fxn*-cKO males (with floxed *Fxn* at the exon 2) started to die significantly earlier compared to *Fxn*-cKO females, as shown in Kaplan-Meier survival curve in Fig. 1b. A total of 20 *Fxn*-cKO mice were included in the survival study: 10 males and 10 females. As shown Fig. 1b, the first death in *Fxn*-cKO males occurred at day 61, while no death was observed in *Fxn*-cKO females until day 76 of the study. This 15-day delay in the onset of death between males and females was significant (P < 0.05). Similarly, most *Fxn*-cKO males died between day 65 and 76, while the majority of females died between day 80 and 89. When we calculated the median survival at the 50% survival point, there was a 10-day shift in survival between males and females: with half of males dying at 71.5 days and half of females dying at 81.5 days of the study. As this difference between males and females has not been reported previously, it was important to determine the underlying mechanisms for the observed findings. *Fxn*-cKO males also stopped gaining weight at week 5 as compared to their littermate controls while *Fxn*-cKO females continued to gain weight until week 8 (Fig. 1c). After 8 weeks both males and females began to lose weight dramatically, with all males dying prematurely by day 84.

### Cardiac structure and function were impacted more severely in *Fxn*-cKO males

Since *Fxn*-cKO males were dying earlier as compared to *Fxn*-cKO females, we designed a cross-sectional study where both males and females were sacrificed at the same time point (8 weeks of age, i.e., at 56 days of age before severe weight loss was observed as shown in Fig. 1c). To examine changes in cardiac structure, we recorded echocardiography (echo) images in the parasternal short axis at the level of the papillary muscles in both CTRL and *Fxn*-cKO males and females. As shown in Fig. 1d, B-mode short axis echo images obtained at diastole revealed that both endocardial and epicardial area of *Fxn*-cKO animals were significantly increased in size as compared to their corresponding controls. Measurement of the epicardial area in all 4 groups revealed that the hearts of *Fxn*-cKO males increased in size by 45%, while the heart size of *Fxn*-cKO females only showed ~30% increase over CTRL females (Fig. 1e). These data corroborate with the heart weights measurements obtained postmortem. As shown in Fig. 1f, *Fxn*-cKO male mice exhibited increased heart weights relative to the CTRL mice (162±7 mg *vs* 107±4 mg), which we similarly found in *Fxn*-cKO female mice compared to their CTRL (131±5 mg vs 88±4 mg). Since a significant drop in the body weights was observed in *Fxn*-cKO males (and not in females) in the cross-sectional study at 8 weeks of age (Fig. 1g), heart weights were normalized to tibial lengths (HW/TL). As shown in Fig. 1h, HW/TL increased 53% in *Fxn*-cKO males as compared to CTRL males (9.16±0.38 mg/mm *vs* 6.01±0.24 mg/mm, p < 0.0001), and 49% in *Fxn*-cKO females as compared to healthy females (7.56±0.33 mg/mm *vs* 5.1±0.19 mg/mm, p < 0.0001).

**Fig. 2:**
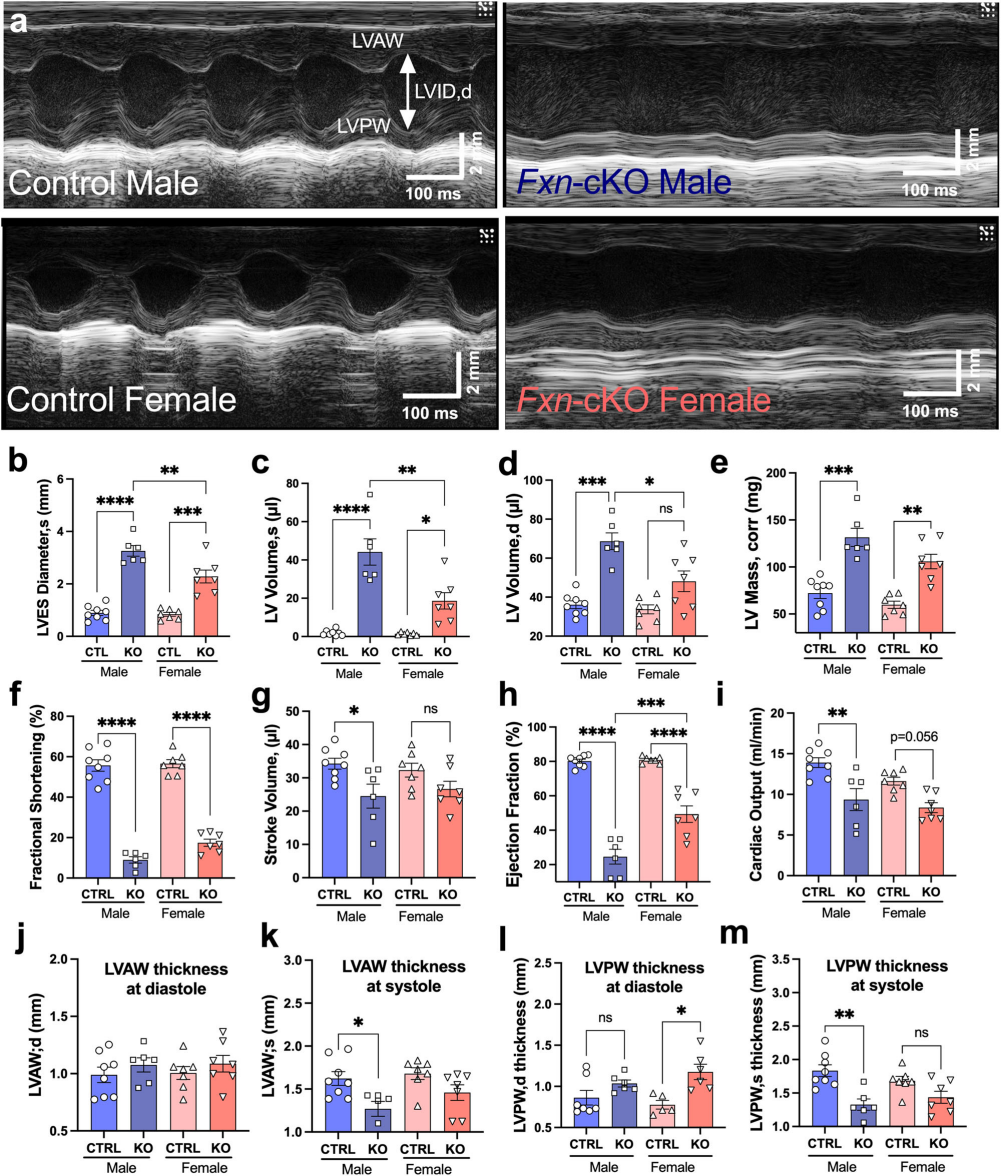
Cardiac function was severely impaired in *Fxn*-cKO mice. **a** Representative M-mode echocardiography images recorded in control (CTRL) and *Fxn*-cKO mice. **b** Left ventricular end-systolic (LVES) diameter (mm). **c** LV Volume, systole (µl). **d** LV Volume, diastole (µl). **e** LV mass, corrected (mg). **f** Fractional shortening (%). **g** Stroke volume (µl). **h** LV Ejection fraction (%). **i** Cardiac output (ml/min). **j** Left ventricle anterior wall (LVAW) thickness in diastole (mm). **k** LVAW thickness in systole (mm). **l** Left ventricle posterior wall (LVPW) thickness in diastole (mm). **m** LVPW thickness in systole. Data presented as a mean ± SEM for CTRL males (n = 8), *Fxn*-cKO males (n = 6), CTRL Females (n = 7), *Fxn*-cKO females (n = 7). Significance was determined by two-way ANOVA with *p < 0.05, **p < 0.01, ***p < 0.001, and ****p < 0.0001. NS – non-significant.

To measure cardiac function, we acquired echo images at the parasternal short axis using M-Mode setting as shown in Fig. 2a. A striking difference was noted in the systolic function and LV dimensions of *Fxn*-cKO hearts as compared to their healthy CTRL. The summary data for LV dimensions in panels 2b-e indicate that the LV diameter at systole increased 3.7-fold in *Fxn*-cKO males (3.26±0.49 *vs* 0.89±0.28 mm) and only 2.7-fold in *Fxn*-cKO females (2.28±0.65 *vs* 0.84±0.19 mm) relative to their respective CTRL (Fig. 2b). Similarly, the volume of LV at systole increased from 1.85±0.49 µl in CTRL males to 44.15±6.88 µl in *Fxn*-cKO males (24-fold increase, P < 0.0001) and from 1.43±0.31 µl in CTRL females to 18.62±4.26 µl in *Fxn*-cKO females (13-fold increase, P < 0.01) indicating volume overload in *Fxn*-cKO animals (Fig. 2c). The LV volume at diastole (Fig. 2d) was also increased in both *Fxn*-cKO males and females, but to a lesser degree compared to the LV volume at systole. The calculation of LV mass revealed 83% increase in *Fxn*-cKO males *vs* CTRL males, and 73% increase in *Fxn*-cKO females (Fig. 2e).

Fractional shortening (FS), a parameter used to assess systolic function, declined 75% in *Fxn*-cKO males (56±2.8 in CTRL, n = 6 to 9±1.6% in KO, n = 8, p < 0.0001) and 51% in *Fxn*-cKO females (from 56±1.9 in CTRL to 18±1.7, n = 7, p < 0.0001) (Fig. 2f). Stroke volume was decreased significantly only in *Fxn*-cKO males (-29%, from 34.3±4.6 µl in CTRL to 24.5±8.9 µl in KO, p < 0.05), while *Fxn*-cKO females only showed a trend towards a decline in stroke volume (-17%, NS) (Fig. 2g). Moreover, as shown in Fig. 2h, *Fxn*-cKO males exhibited a ~ 70% lower ejection fraction (EF) relative to their CTRL (24.6±10.5% vs 80.1±3.3%, p < 0.0001), while *Fxn*-cKO females only showed a ~ 39% decrease (49.3±12.6% vs 80.9±1.9%, p < 0.0001). Similarly, cardiac output was decreased by 33% in *Fxn*-cKO males (p < 0.0001), with only 10% decrease (p = 0.056) observed in *Fxn*-cKO females (Fig. 2i).

The LV anterior wall (LVAW) thickness at diastole was not changed significantly in any group (Fig. 2j), however there was a significant decrease (p < 0.05) in LVAW thickness in systole (Fig. 2k). LV posterior wall (LVPW) thickness at diastole was increased by 51% in *Fxn*-cKO females (Fig. 2l), while LVPW thickness at systole was decreased by 28% only in *Fxn*-cKO males (Fig. 2m) indicating dilatation of heart wall in *Fxn*-cKO males. Altogether, these data indicate that *Fxn*-cKO males had a significantly higher decline in cardiac function, with LV EF decreasing almost two times more in *Fxn*-cKO males as compared to *Fxn*-cKO females.

### There were no significant differences in frataxin expression in male and female mice

**Fig. 3:**
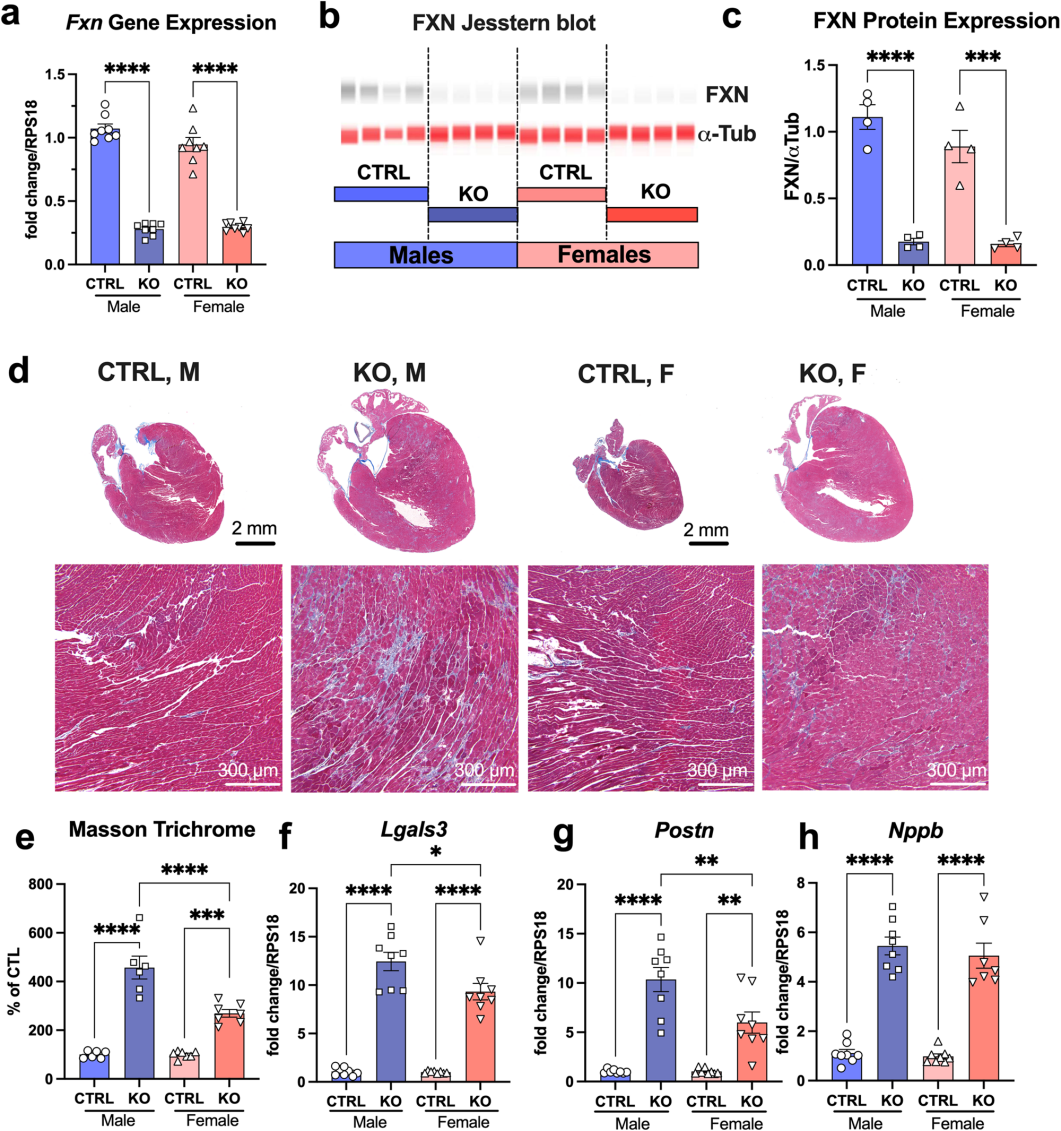
Frataxin decline in the heart correlated with the impairment in cardiac function and fibrosis development. **a** *Fxn* gene expression in the hearts of 8 weeks old mice. n = 8 for each group for gene expression. **b** Representative Jesstern blot images for frataxin (FXN) and α-tubulin protein expression in whole heart homogenates. **c** Jesstern blot summary for FXN protein expression normalized to α-tubulin. n = 4 for each group for protein expression. **d**. Representative images of control and *Fxn*-cKO hearts stained with Masson trichrome for fibrotic tissue detection. **e** Masson trichrome summary data collected and analyzed from CTRL male (n = 6), KO male (n = 6), CTRL female (n = 7), and KO female (n = 7) longitudinal cross sections of the heart. Data are expressed as a percent of control. **f** Galectin-3 (*Lgals3*) gene expression in the heart. **g** Periostin (*Postn*) gene expression in the heart. **h** Natriuretic peptide type B (*Nppb*) gene expression in the heart. Data presented as mean ± SEM. n = 7–8 for each group for gene expression. In all experiments, n number represents biological replicates of heart samples from the individual mice. Significance was determined by two-way ANOVA with *p < 0.05, **p < 0.01, ***p < 0.001, and ****p < 0.0001.

To directly test if the sexual dimorphism could be explained by a different degree of frataxin knock-out in males *vs* females, we examined frataxin gene and protein expression in the heart tissues collected from CTRL and KO animals. However, both males and females showed a similar degree of frataxin knock-out, with approximately 15% frataxin remaining in the heart as assessed by gene (Fig. 3a) and protein (Fig. 3b, c and Supplementary Fig. 1) expression. These experiments were performed in the whole-heart extracts, which could have contributions from non-cardiomyocyte cells that did not contain MCK and therefore did not experience *Fxn* knockout.

To verify the presence of non-cardiomyocyte cells (i.e., fibroblasts) in both CTRL and KO hearts, fibrosis development was examined by Masson Trichrome staining, which showed collagenous connective tissue fibers in blue, and muscle fibers in red (Fig. 3d). In agreement with data presented in Fig. 1d–h, KO hearts exhibited significant hypertrophy, which was more pronounced in male KO hearts as compared to KO females (Fig. 3d). Furthermore, the percentage of fibrotic tissue was significantly increased in KO hearts, with males showing 467% more fibrosis relative to CTRL male hearts (p < 0.0001), while *Fxn*-cKO females showing only a 267% higher fibrosis compared to CTRL female hearts (p < 0.001) (Fig. 3e). Gene markers of activated fibroblasts, and therefore fibrosis such as *Lgals3*, which encodes Galectin 3 (Fig. 3f) and *Postn*, which encodes periostin (Fig. 3g), were also elevated significantly higher in *Fxn*-cKO males compared to KO females. At the same time, heart failure marker *Nppb*, which encodes B-type natriuretic peptide (BNP), a hormone secreted primarily by the ventricular myocardium in response to wall stress such as volume expansion and pressure overload, was elevated to a similar degree in both males and females (Fig. 3h).

In summary, these data indicate that both *Fxn*-cKO male and female mice developed dilated cardiomyopathy with decreased ejection fraction at 8 weeks of age. However, the severity of cardiac dysfunction and structural changes were more pronounced in *Fxn*-cKO male mice relative to *Fxn*-cKO female mice, which could explain their premature death in survival studies as shown in Fig. 1b.

### Kidney size and function were decreased more severely in *Fxn*-cKO males

**Fig. 4:**
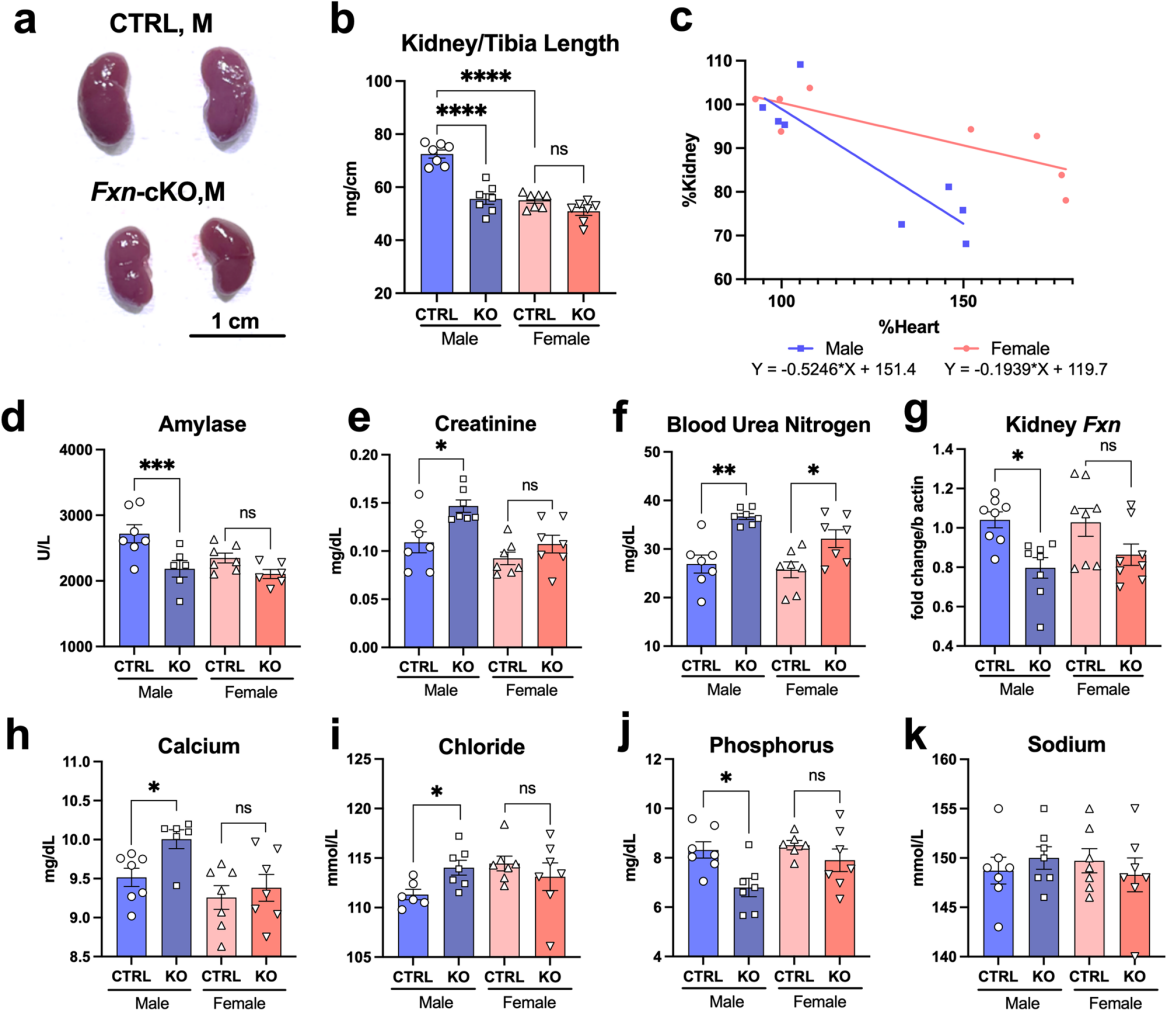
Kidney size and function were decreased more severely in *Fxn*-cKO males. **a** Representative image of kidneys from control and *Fxn*-cKO males. **b** Kidney weight/tibia length (mg/cm) at eight weeks of age. Average male kidney weights measurements: CTRL male= 70.4 *vs* KO male 52.4 mg/cm; CTRL female =56.12 *vs* KO female = 48.9 mg/cm. **c** Correlation between percent of kidney weight as a function of percent of heart weight. Male regression: y = -0.5246x + 151.4; Female y = -0.1939x + 119.7. **d** Serum amylase levels. **e** Serum creatinine levels. **f** Serum blood urea nitrogen levels. **g** Kidney *Fxn* gene expression. **h** Serum calcium levels. **i** Serum chloride levels. **j** Serum phosphorus levels. **k** Serum sodium levels. Data presented as mean ± SEM with n = 8 for each group. In all experiments, n number represents biological replicates of kidney or serum samples collected from the individual mice in each group. Significance was determined by two-way ANOVA with *p < 0.05, **p < 0.01, ***p < 0.001, ****p < 0.0001. NS – non-significant.

In the survival cohort animals, we observed the development of significant abdominal edema in approximately 40% of our KO animals, which led us to examine kidney function in *Fxn*-cKO animals. During dissection, we noted that kidneys of *Fxn*-cKO males were severely atrophied (Fig. 4a). Kidney weight normalized to tibial length showed a 23% decrease in kidney size in *Fxn*-cKO males as compared to control male littermates, while KO females showed a non-significant 7% decrease in kidney size (Fig. 4b). While comparing hearts and kidneys as a percentage of healthy controls, it is evident that with a similar increase in heart size in both males and females, males experienced a much more severe decrease in kidney weight (Fig. 4c), with a linear regression showing a slope twice as steep compared to females and R^2^ value of 0.78 and 0.69, respectively. Kidney serum biochemistry profiling was performed in these mice and revealed a significant decrease in circulating amylase in *Fxn*-cKO males (Fig. 4d). At the same time there was a significant increase in creatinine (Fig. 4e) and blood urea nitrogen (BUN) (Fig. 4f) in *Fxn*-cKO males. BUN was also elevated in *Fxn*-cKO females (Fig. 4f) and there was a non-significant trend towards increase in creatine level in females (Fig. 4e).

While poor cardiac health can impact kidney function, the kidney dysfunction appears to be greater than that could be caused by renal congestion due to heart failure. It has not been previously reported that these mice experience kidney damage, or any FXN loss in the kidneys. We have found that both MCK and FXN are also expressed in the ascending loop of Henle in the nephron^19,20^, indicating that this is a location that would experience a knockout of frataxin. The ascending loop of Henle is responsible for both passive and active reabsorption of sodium and chloride from the urine. The thick ascending loop of Henle is rich in mitochondria, and in addition to sodium and chloride reabsorption, large amounts of potassium, calcium, and magnesium are reabsorbed in this region of nephron in an energy-efficient manner^21^. Therefore, a decrease in FXN expression in the ascending loop of Henle could affect nephron’s function and electrolyte balance in the blood. We examined *Fxn* gene expression in the kidneys and determined that there was a small (~24%) but significant decrease in *Fxn* gene expression in *Fxn*-cKO males, while *Fxn* decrease in females was not significant (Fig. 4g). Consistent with impaired kidney function, we found a significant increase in concentration of circulating calcium (Fig. 4h), and chloride (Fig. 4i) in *Fxn*-cKO males as compared to their control littermates, while circulating phosphorus concentration was significantly decreased (Fig. 4j). No significant change was observed in serum sodium levels in males or females (Fig. 4k). No significant differences in circulating ions and anions were observed in *Fxn*-cKO females (Fig. 4h–k). Greater kidney damage in *Fxn*-cKO males compared to KO females could partially explain the early onset of death in males, but still does not completely explain why *Fxn*-cKO males are being affected more severely than females.

### Differences in steroidogenesis and cholesterol transport in *Fxn*-cKO males and females

**Fig. 5:**
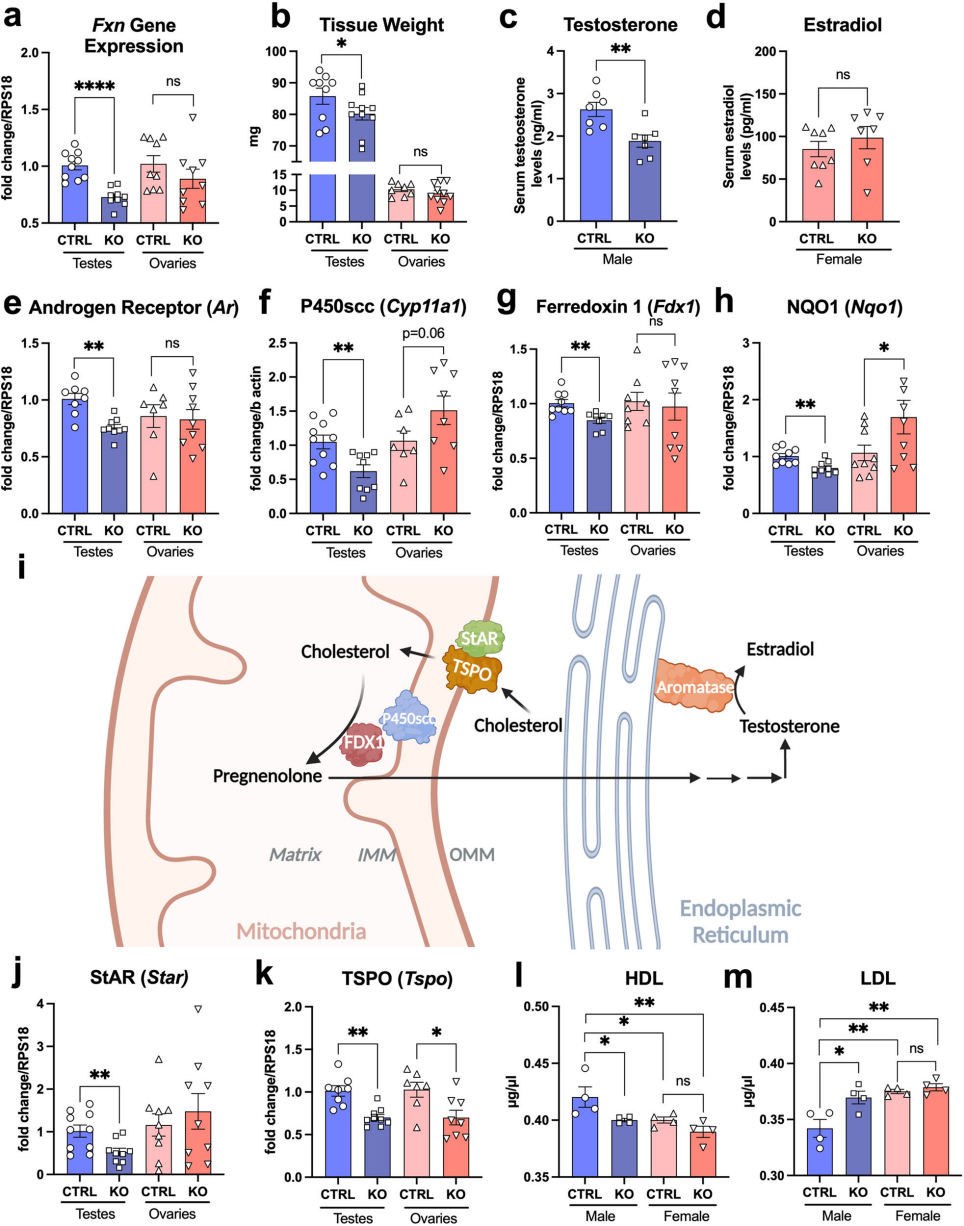
Differences in steroidogenesis and cholesterol transport in testes and ovaries of *Fxn*-cKO mice. **a** *Fxn* gene expression in testes and ovaries in control and *Fxn*-cKO mice. **b** Testes and ovaries weight. **c** Serum testosterone levels. **d** Serum estradiol levels. **e** Androgen receptor (*Ar*) gene expression. **f** Cytochrome P450scc (*Cyp11a1*) gene expression. **g** FDX1 (*Fdx1*) gene expression. **h** NQO1 (*Nqo1*) gene expression. **i** Schematic representation of cholesterol conversion to testosterone and estradiol. Created with BioRender.com **j** StAR (*Star*) gene expression. **k** TSPO (*Tspo*) gene expression. **l** Serum high-density lipoprotein (HDL) cholesterol levels in male and female mice. **m** Serum low-density lipoprotein (LDL) cholesterol levels in male and female mice. Data presented as mean ± SEM. n = 7-10 per group for gene expression. n = 4 per group for serum HDL and LDL. The symbols in each panel represent the number of individual mice (defined as biological replicates) involved in this study. Significance was determined by one-way ANOVA with *p < 0.05 **p < 0.01 ***p < 0.001. NS non-significant.

Another location where FXN and MCK proteins appear to be expressed together is the male reproductive system, in both the Sertoli cells and Leydig cells, which are responsible for spermatogenesis and testosterone/estrogen production in males, respectively^22^. Similarly, MCK and FXN are both expressed in Theca cells of the ovarian follicles in females, where estrogen production occurs^20^. We, therefore, verified *Fxn* gene expression in testes and ovaries of *Fxn*-cKO animals. These data are shown in Fig. 5a and demonstrate that that there was a significant 27% decrease in *Fxn* expression in testes of *Fxn*-cKO males as compared to CTRL littermates, with no significant changes observed in *Fxn* gene expression in ovaries of the *Fxn*-cKO females.

Furthermore, *Fxn*-cKO males showed ~10% decrease in testicle weight as compared to CTRL mice (Fig. 5b), which often correlates with testosterone loss in KO males. Indeed, a significant 28% decrease in circulating testosterone level was detected in *Fxn*-cKO males as compared to healthy CTRL males (Fig. 5c). Next, we examined the ovary weight in *Fxn*-cKO females which showed no significant differences as compared to CTRL females (Fig. 5b), and as expected, no significant changes observed in circulating estrogen levels (Fig. 5d). In agreement with these data, we found that expression level of androgen receptor was decreased by 25% in testes of KO males (Fig. 5e), with no changes observed in expression of estrogen receptor 2 (ESR2) in *Fxn*-cKO females (Supplementary Fig. 2a).

The first step of both testosterone and estrogen synthesis occurs in the inner mitochondrial membrane converting cholesterol to pregnenolone by the cholesterol side-chain cleavage enzyme cytochrome P450 (P450scc), which is encoded by the *Cyp11a1* gene (Fig. 5f, i). P450scc enzyme requires a transfer of 2 pairs of electrons, aided by ferredoxin 1 (FDX1) which contains iron-sulfur clusters (Fig. 5i)^23^. As shown in Fig. 5f, testicular and ovarian gene expression of *Cyp11a1* showed a 41% decrease in KO males, and conversely, ~42% increase in cKO females. Next, we examined expression of aromatase (estrogen synthase), which is a key enzyme in estrogen biosynthesis^24^. This enzyme is encoded by *Cyp19a1* gene and is responsible for the aromatization of androgens into estrogens. There was no difference in *Cyp19a1* gene expression in either KO males or KO females (Supplementary Fig. 2b). Expression of FDX1 (*Fdx1*) was decreased by 16% in testes of *Fxn*-cKO males (Fig. 5g), which was also associated with a 21% decrease in NAD(P)H quinone oxidoreductase (*Nqo1*) gene expression (Fig. 5h). This enzyme plays a major role in the regulation of oxidative stress and its silencing is reported to aggravate hormone-induced damage to the reproductive organs in mice^25^. A similar trend was observed in the expression of the glutathione S-transferase pi 1 (GSTP1, *Gstp1* gene), which catalyzes the conjugation of glutathione (GSH) to a wide variety of endogenous and exogenous electrophilic compounds and protects cellular macromolecules from attack by reactive electrophiles. We observed a ~15% decrease in GSTP expression only in testes of *Fxn*-cKO males, but not in the ovaries of KO females (Supplementary Fig. 2c).

Before pregnenolone formation in mitochondrial matrix could occur, cholesterol needs to be transferred from the outer mitochondrial membrane to the inner mitochondrial matrix (Fig. 5i) by the action of the steroidogenic acute regulatory protein (StAR), which binds cholesterol and its subsequent transport by the 18-kDa translocator protein of the outer mitochondrial protein (TSPO, formerly known as a peripheral benzodiazepine receptor)^26^. We, therefore, examined the expression levels of StAR and TSPO and found that *Star* gene expression was decreased in testes of *Fxn*-cKO males by 48% (Fig. 5j), while *Tspo* gene expression was decreased in both testes and ovaries of *Fxn*-cKO animals by ~30% (Fig. 5k).

Next, serum levels of high-density (HDL) (Fig. 5l) and low-density lipoprotein (LDL) (Fig. 5m) cholesterol were measured in both males and females. Our data revealed that HDL levels were significantly decreased only in *Fxn*-cKO males. We noticed that in general HDL levels were lower in females as compared to males. At the same time, serum LDH levels were significantly increased in *Fxn*-cKO males (Fig. 5m). It has been previously shown that testosterone-deficient male mice had significantly increased serum cholesterol levels^27,28^, which could contribute to the severity of the observed cardiac dysfunction in *Fxn-*cKO males. As vitamin D is a precursor to both testosterone and estrogen, circulating vitamin D levels were measured, with no change found in both *Fxn*-cKO males and females (Supplementary Fig. 2d). These data suggest that testosterone decline was directly linked to Leydig cells dysfunction, and not a vitamin D deficiency. In summary, we concluded that in addition to the heart, FXN expression was significantly decreased in kidneys and testes of *Fxn*-cKO males, which was associated with imbalance of circulating ion/anions concentrations (Fig. 4) and a significant decrease in testosterone production in *Fxn*-cKO males. Interestingly, we found that *Esr1* gene expression of estrogen receptor ERα (ESR1) in the heart was elevated in both *Fxn*-cKO males and females (Supplementary Fig. 2e), but it was significant only in males suggesting that cardiac signaling processes could be affected by estrogens in FXN-cKO males.

### Key players in cardiac ECC were more severely affected in *Fxn*-cKO males

In men, testosterone levels begin to decrease after age 40, and this decrease has been associated with an increase in all-cause mortality and cardiovascular (CV) risk^29^. Similarly, it is known that pre-menopause women are protected from cardiac disease by circulating estrogens^30^.

**Fig. 6:**
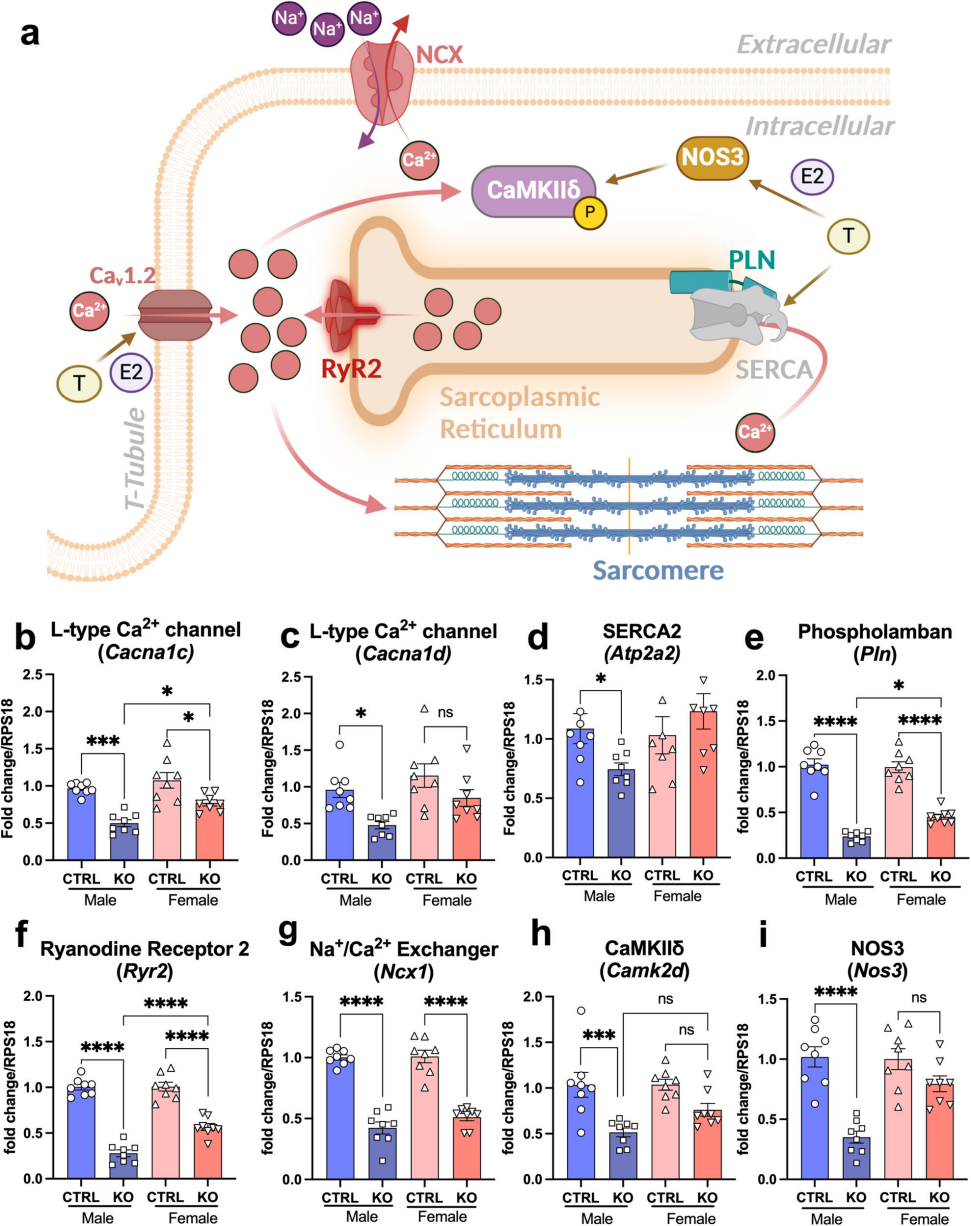
Key players in cardiac excitation-contraction coupling system were more severely affected in *Fxn*-cKO males. **a** Schematic representation of possible effects of testosterone and estrogen on calcium signaling in the heart. Created with BioRender.com **b** L-type Ca^2+^ channel subunit alpha1 C (CaV1.2) gene (*Cacna1c*) expression in control and *Fxn*-cKO male and female hearts. **c** L-type Ca^2+^ channel subunit alpha1 D (CaV1.3) gene (*Cacna1d*) expression. **d** SERCA2 (*Atp2a2*) gene expression in the heart. **e** Phospholamban (*Pln*) gene expression. **f** RyR2 (*Ryr2*) gene expression in the heart. **g** NCX1 (*Ncx1*) gene expression in the heart. **h** CaMKIIδ (*Camk2d*) gene expression in the heart. **i** NOS3 (*Nos3*) gene expression in the heart. Data presented as mean ± SEM, n = 8 animals for each group. Significance was determined by two-way ANOVA with *p < 0.05, ***p < 0.001, and ****p < 0.0001. NS non-significant.

Disrupted cardiomyocyte calcium ions (Ca^2+^) homeostasis is recognized as a major contributor to the development of congestive heart failure phenotype^31^. Both estrogen and testosterone can impact L-type calcium channels located on the surface of cardiomyocytes and responsible to bring Ca^2+^ inside to initiate the proper process of cardiac excitation-contraction coupling (Fig. 6a)^32^. Indeed, we found that *Fxn*-cKO males showed a significant decrease in *Cacna1c* (48% decrease) and *Cacna1d* (50% decrease) gene expression, which encode the calcium voltage-gated channel subunit alpha1 C (Ca_V_1.2) and calcium voltage-gated channel subunit alpha1 D (Ca_V_1.3), respectively (Fig. 6b, c). In *Fxn*-cKO females*, Cacna1c* (Ca_V_1.2) was decreased by 33% and there was a non-significant decrease in *Cacna1d* (-26%) gene expression. However, *Atp2a2* gene expression levels of Ca^2+^-ATPase of the sarcoplasmic reticulum (SERCA2), which is responsible for Ca^2+^ re-uptake back to the sarcoplasmic reticulum following Ca^2+^ transient release during each heartbeat (and, therefore, a proper relaxation of the heart during diastole) was decreased by 35% only in *Fxn*-cKO males (Fig. 6d). In accordance with a decrease of SERCA2, phospholamban (*Pln*) gene expression was also decreased in both male and female *Fxn*-cKOs, with a greater decrease in males (-77% in males, -57% in females) (Fig. 6e). Furthermore, cardiac *Ryr2* gene expression of the Ryanodine receptor 2 (RyR2) (Fig. 6f) was decreased by 73% in *Fxn*-cKO males with a smaller decrease observed in KO females (-44%). In contrast to the reported data for pressure-overload-induced heart failure and dilated cardiomyopathy, where Na^+^/Ca^2+^ exchanger 1 (NCX1) and Ca^2+^-calmodulin-dependent protein kinase II delta (CaMKIIδ) are typically upregulated^33,34^, we found that both *Ncx1* and *Camk2d* gene expression was significantly decreased in both *Fxn*-cKO males (–58% for *Ncx1* and -49% *Camk2d*) and females (–50% for *Ncx1* and -25% for *Camk2d*) (Fig. 6g, h).

Both estrogen and testosterone could affect nitric oxide production in the heart, kidneys, and blood vessels, which would subsequently impact cardiac repolarization, vasodilation, and signaling via cGMP/protein kinase G pathway. We found that expression of the *Nos3* gene, which encodes endothelial nitric oxide synthase 3 (NOS3), was decreased in *Fxn*-cKO males by 65%, but only showed a trend to decrease in females (Fig. 6i), further emphasizing a testosterone-mediated difference in contributing to worse kidney function, worse cardiac function, and therefore overall survival. Altogether, these data indicate that cardiac and kidney function was more severely affected in *Fxn*-cKO males potentially due to decline in testosterone production, its impact on cardiac-excitation contraction coupling and the corresponding decline in contractile function.

### No sexual dimorphism is observed in oxidative phosphorylation in hearts of *Fxn*-cKO mice

**Fig. 7:**
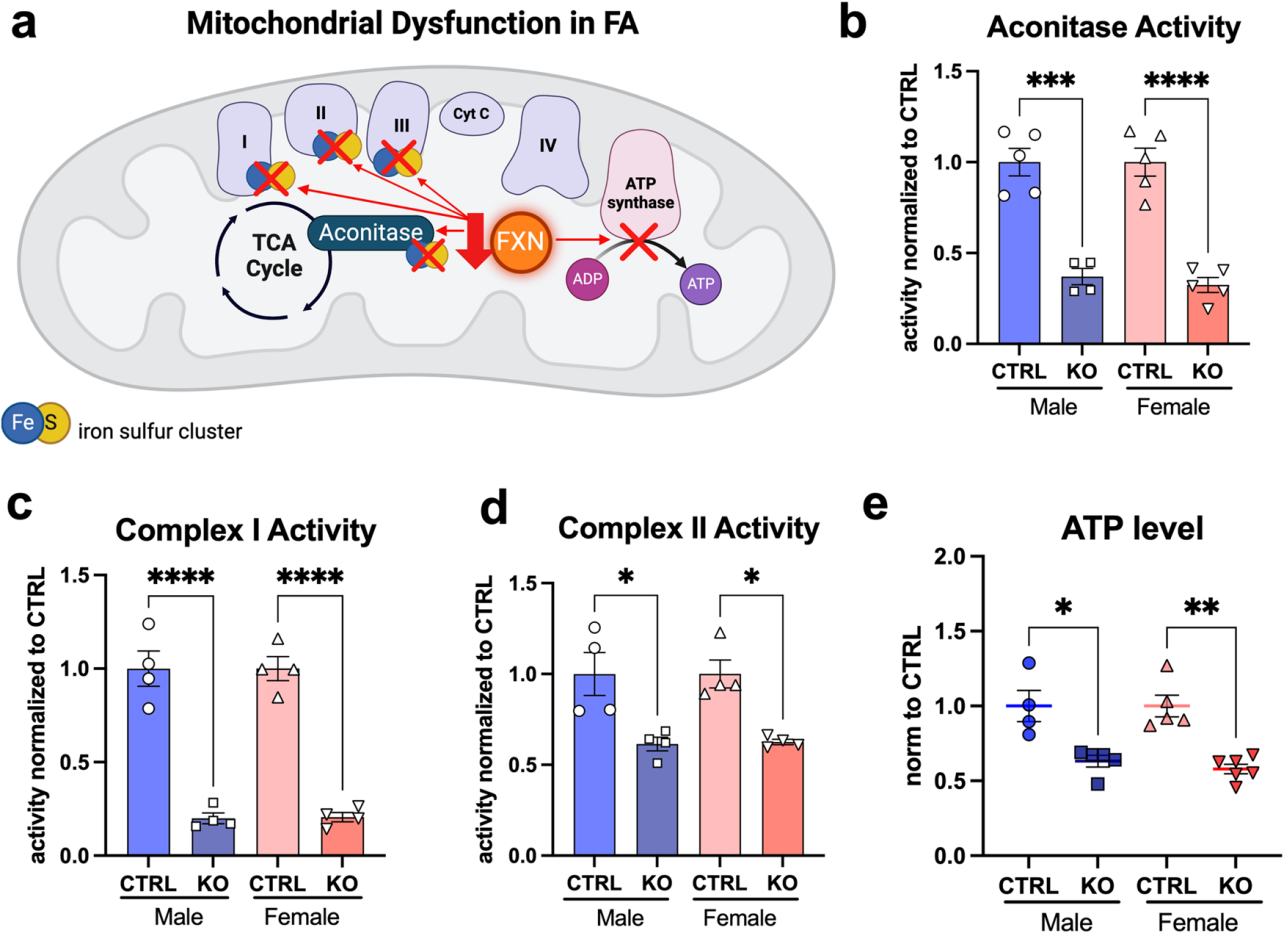
Mitochondrial dysfunction in FA. **a** Schematic cartoon representing mitochondrial dysfunction in FA. Created with BioRender.com. **b** Aconitase activity measured in control and *Fxn-cKO* hearts. **c** Mitochondrial complex I activity in control and *Fxn-cKO* hearts. **d** Mitochondrial complex II activity in heart tissue from control and Fxn-cKO mice. **e** ATP levels in heart tissue from control and *Fxn-cKO* mice. Data presented as mean ± SEM, n = 4-5 individual heart samples for each group. Significance level was determined by two-way ANOVA with *p < 0.05, **p < 0.01, ***p < 0.001, and ****p < 0.0001.

Since FA is a mitochondrial disorder, we examined if there was a difference in mitochondrial function in *Fxn*-cKO hearts. The loss of iron sulfur clusters formation in FA impairs the electron transport chain and aconitase activity in mitochondria (Fig. 7a). Indeed, we confirmed that aconitase activity was decreased in FXN-cKO hearts, however the degree of the impairment was similar (~65% decline in activity) in both male and female *Fxn*-cKO mice (Fig. 7b). A recent study has revealed that FXN interacts directly with the mitochondrial respiratory complexes I, II, and III^35^. We found that mitochondrial complex I and II activities were decreased to a similar degree in both *Fxn*-cKO male and female mice (~80% decrease in complex I activity and ~40% decreased in complex II activity) (Fig. 7c, d). Due to decreased activity of aconitase and mitochondrial complexes I and II, ATP levels were also decreased by ~60% in *Fxn*-cKO mice with no sexual dimorphism observed between KO males and females (Fig. 7e). These findings are not surprising as FXN expression was equally decreased in both male and female *Fxn*-cKO hearts as shown in Fig. 3a–c; and therefore, the loss of iron-sulfur clusters affects the mitochondrial respiratory chain complexes activity and ATP production similarly.

## Discussion

The *Fxn*-cKO mice represent a model of end-stage cardiomyopathy in FA. Loss of FXN in the heart causes dilated left ventricular hypertrophy, ultimately resulting in death due to HF in these mice. This study provides evidence of sexual dimorphism in the mouse model of FA with more pronounced deficits in cardiac, renal, and reproductive system function in *Fxn*-cKO males. First, *Fxn*-cKO males started to die ~2 weeks earlier as compared to *Fxn*-cKO females, surviving a median of 10 days fewer than females. In a model where lifespan is already short, this is a proportionally larger difference if the calculations are made in terms of human life (Fig. 1). *Fxn*-cKO males exhibited lower cardiac ejection fraction, fractional shortening, and stroke volume compared to KO females, as well as a greater increase in LV diameter (Figs. 1, 2). With no difference in the residual frataxin levels, or major HF markers, we sought to investigate the mechanisms underlying the differences in cardiac function and life expectancy in *Fxn*-cKO males and females.

We determined that *Fxn*-cKO males exhibited a significantly lower level of circulating testosterone as compared to their healthy littermates (Fig. 5c) while KO females were able to maintain estrogen levels (Fig. 5d). Furthermore, gene expression of several proteins involved in the process of excitation-contraction coupling, especially expression of L-type calcium channels, RyR2 and SERCA was decreased significantly more in *Fxn*-cKO males (Fig. 6). Decreased expression of L-type calcium channels can negatively impact excitation-contraction coupling and potentially contribute to cardiac arrhythmias. Indeed, a decrease in Ca_V_1.3 expression has been shown to cause sinoatrial node dysfunction^36^. Other studies showed that Ca_V_1.2 expression directly correlated with testosterone levels, and that supplementation with testosterone increased expression of Ca_V_1.2 and Ca_V_1.3 L-type calcium channels^22,37^. CaMKIIδ gene expression was decreased in both male and female KO mice, which also indicates further dysregulation in Ca^2+^ signaling. Interestingly, Esr1 gene which encodes estrogen receptor alpha (ESR1), was upregulated in male *Fxn*-cKO hearts but not in females (Supplementary Fig. 2e). ESR1 has been shown to be upregulated in dilated cardiomyopathy, likely as a compensatory response, as ERα can aid in myocardial protection^38,39^. Furthermore, it has been shown that estrogens could have a cytoprotective effect in human skin FA fibroblasts via non-genomic mechanisms^40^. This effect was independent from ERα activation and likely mediated by the ability of phenolic estrogens to act as antioxidants, causing scavenging of reactive oxygen species.

This mouse model relies on the Cre promoter to excise the *Fxn* gene only in the presence of MCK. Knockout of FXN in these target tissues during development would result in embryonic lethality as these tissues would be unable to develop. Therefore, the actual knockout of FXN is likely progressive. At three weeks of age, no morphological differences were observed in the hearts or kidneys of KO animals *vs* corresponding controls of either sex (Supplementary Fig. 3). Similarly, serum markers of kidneys function were not different in KO mice as compared to CTRL animals at 3 weeks of age (Supplementary Fig. 3). However, we observed a significant difference in kidneys size at 8 weeks of age. The kidneys of *Fxn*-cKO males appeared significantly atrophied, whereas KO female kidneys were only slightly smaller. In addition, serum markers of kidney damage were significantly elevated only in *Fxn*-cKO males and not in KO females (Fig. 4). Both MCK and FXN are expressed in the ascending loop of Henle (LOH) in the kidneys, which could cause a partial knockout of FXN at that region. Partial knockout of FXN in the LOH could cause deficits in sodium and chloride exchanges in the LOH and lead to the increase in concentration of circulating sodium and chloride. Indeed, as shown in Fig. 4i serum chloride level was significantly elevated in *Fxn*-cKO males (and not in KO females), and there was a non-significant trend towards sodium elevation in KO males (Fig. 4k). There was also a significant increase in circulating calcium concentration (Fig. 4h) and decrease in serum phosphorus levels (Fig. 4j) in *Fxn*-cKO males. This could be an explanation for the kidney damage in these animals and the appearance of the abdominal edema near the end of life.

Clinical data on kidney dysfunction in FA are very limited. We only found a couple of clinical reports describing several FA patients presented with the nephrotic syndrome (NS)^41,42^ or kidney infarction^43^. NS is a kidney disorder defined by hyperproteinemia that causes your body to pass too much protein in the urine. It is a rare idiopathic disease mostly observed in pediatric patients and associated with the damage in kidney’s blood-filtering units called glomeruli leading to hyperproteinemia, hyperlipidemia, and edema. The exact cause of NS is unknown; however, it was shown that treatment of these patients with steroids to ameliorate symptoms of NS was also improving neurological symptoms of FA.

The most convincing evidence of kidney dysfunction in FA was presented as part of the clinical MOXIe trial (NCT02255435) characterizing effects of omaveloxelone (OMAV) on neurological function in patients with FA^44^. During this trial, effects of OMAV on kidney function in patients with FA were evaluated^45^. In this study, 103 patients between 16 and 40 years of age with genetically confirmed FA were randomly split in placebo and OMAV treated groups administered once daily for 48 weeks. This study revealed that FA patients in placebo-treated group had a significant decline in the estimated Glomerular filtration rate (eGFR) over 48 weeks that was similar to the eGFR decline observed in the most rapidly progressing form of chronic kidney disease. This study also included a small group of pediatric FA patients (24 individuals) in which the rate of eGFR decline was even more pronounced as compared to adult patients. OMAV treatment improved eGFR in both adult and pediatric patients. Altogether, these data indicate that there is a connection between frataxin decline and kidney dysfunction in FA patients. We are the first group to report kidney dysfunction in FXN-cKO males and its correlation with decreased levels of testosterone.

MCK and FXN are also expressed in both Sertoli and Leydig cells, which are present only in males. These cells are involved in spermatogenesis and testosterone production, respectively. Testosterone is synthesized in Leydig cells, with the first step occurring in the mitochondria^23–26^. With a loss of frataxin in those mitochondria, there was an impairment in testosterone production causing males to produce less testosterone. Low testosterone is linked to both poor kidney outcomes, as well as poor cardiac outcomes. Females received cardiac protection from circulating estrogen that is known to protect muscle membrane from injury^30,46^.

**Fig. 8:**
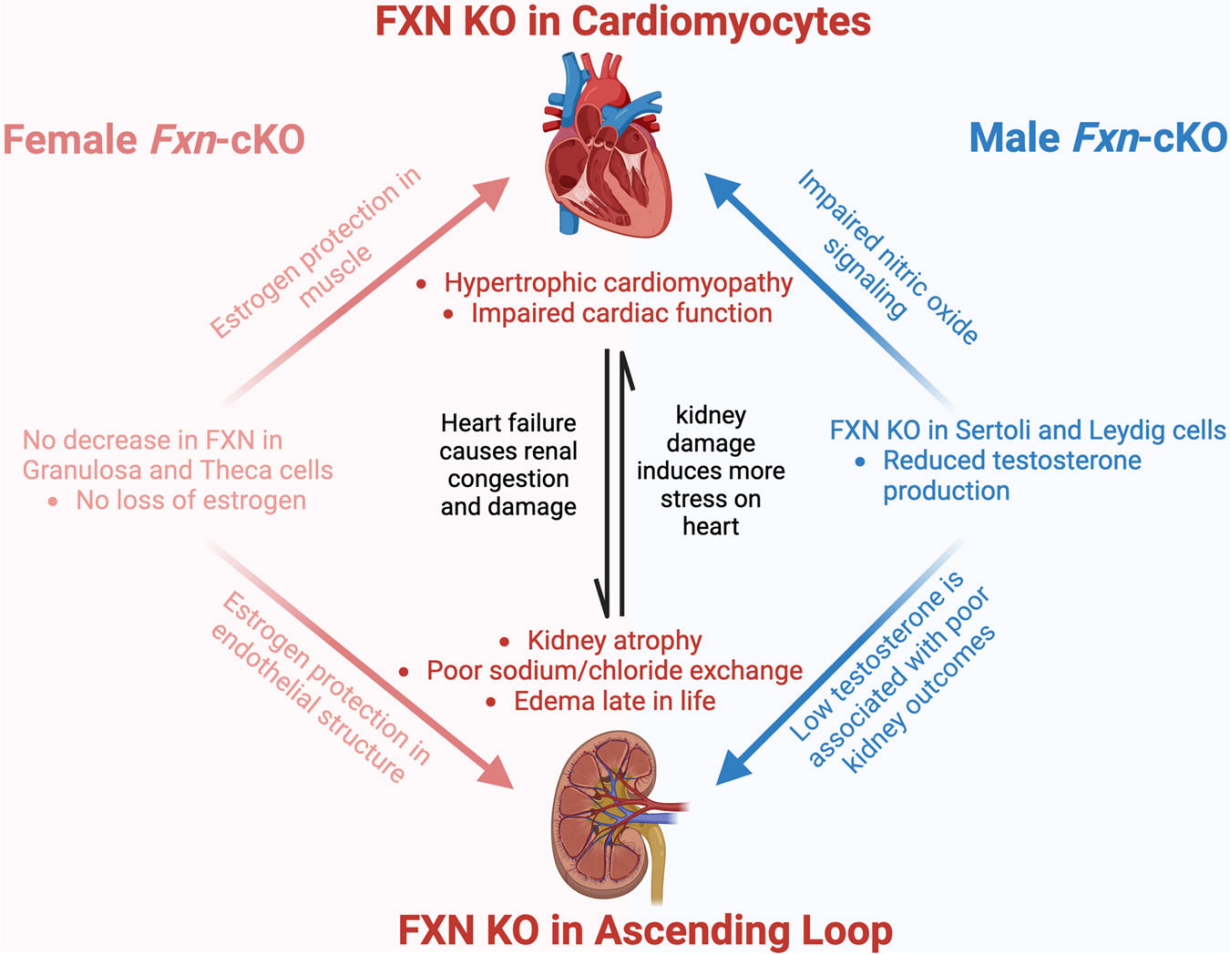
Schematic representation of the interplay between heart, kidneys and reproductive organs in male and female *Fxn-*cKO mice. Created with BioRender.com.

Indeed, hormonal deficiencies can cause or exacerbate organ damage (Fig. 8). As stated above, both testosterone and estrogen can protect structure and function in both heart and kidneys due to nitric oxide signaling^47^. Literature on testosterone impacting cardiac function is divided. Some publications indicate that high levels of testosterone are ultimately bad for the heart, but others say too little testosterone is also detrimental to heart function^48^. Therefore, a fine balance in testosterone level is crucial to cardiac function (Fig. 8). In contrast, estrogen has repeatedly been shown to be beneficial and protective to cardiac structure and function^30^. We believe that in KO males, the low level of testosterone could negatively affect the kidneys and heart function, while in females, the estrogen could have a protective effect. This could partially explain the discrepancy seen between *Fxn*-cKO males and females. More studies are required to determine whether testosterone therapy could be protective in male patients with FA. Interestingly, testosterone deficiency has been recognized as one of the major endocrine disorders in chronic kidney disease^49^.

Testosterone is synthesized by the Leydig cells in testes following luteinizing hormone (LH) stimulation^50^. In females, LH stimulates steroid release from the ovaries, ovulation, and the release of progesterone after ovulation by the corpus luteum^51^. LH binds to G protein-coupled receptors, resulting in the activation of adenylyl cyclase, increased intracellular cAMP formation and cAMP-dependent phosphorylation of proteins through protein kinase A (PKA). This process initiates cAMP-activated cholesterol transfer from intracellular stores into mitochondria, with the subsequent conversion of cholesterol to pregnenolone by the C27 cholesterol side-chain cleavage cytochrome P450 enzyme (P450scc encoded by *Cyp11a1*) located on the matrix side of the inner mitochondrial membrane (IMM) (Fig. 5i). Pregnenolone then diffuses passively from mitochondria to the endoplasmic reticulum (ER) where further enzymatic reactions occur to produce the final steroid products including testosterone and estrogen^52^.

It has been shown that the translocation of cholesterol from the outer mitochondrial membrane (OMM) to the IMM is the rate-limiting step in the production of all steroids, which requires cholesterol binding to the steroidogenic acute regulatory protein (StAR)^53^ on the OMM and subsequent transfer to the IMM mediated by the 18-kDa translocator protein (TSPO) of the outer mitochondrial membrane^54^. Gene expression of both StAR (*Star*) protein was decreased in *Fxn*-cKO males but not in females (Fig. 5j), while gene expression of TSPO (*Tspo*) protein was decreased to a similar degree in both *Fxn*-cKO males and females (Fig. 5k). These are two rate-limiting steps which occur in Leydig cells in males and Theca cells in females and are crucial for cholesterol transport from the OMM to the IMM, where the next limiting step involving P450scc occurs^26,55^. P450scc is a mitochondrial cytochrome p450 which requires the transfer of three pairs of electrons from its redox partners to convert cholesterol to pregnenolone. These electrons are transferred from the reduced nicotinamide adenine dinucleotide (NADPH) to the heme on P450scc with assistance of two proteins: ferredoxin reductase (a flavoprotein) and ferredoxin 1 (FDX1, an iron-sulfur protein)^55,56^. This step could be directly linked to the loss of frataxin as frataxin is involved in the persulfide transfer in iron-sulfur clusters^5^. FDX1 is downregulated in testes of *Fxn*-cKO males (Fig. 5g), but not in ovaries of *Fxn*-cKO females. Therefore, decreased expression of FXN in testes could affect FDX1 and StAR expression, decrease cholesterol transport to mitochondria and serve as the main driver for the decline in testosterone production in *Fxn*-cKO males.

More studies are beginning to emerge on sexual dimorphism in mouse models of FA. For example, Fil et al. demonstrated that males show a reduced survival in FXN^G127V/G127V^ mice compared to females^57^. Additionally, they observed sex differences in neurobehavioral testing, showing that females performed worse than males in the Open field maze testing (locomotor activity testing), whereas males performed worse than females during grip strength testing. Sayles et al. discuss some sex differences in transcriptomic analyses in KIKO700 mice and FXN^G127^ mice with the most significant being the activation of mitochondrial integrated stress response in males appearing earlier than females in FXN^G127^ mice^14^.

A recent study by Perfitt et al. used a new cardiac-specific model with floxed *Fxn* allele homozygosity MCK-*Fxn* ^flox-flox^ mouse showing sexual dimorphism in cardiac function^58^. The mouse used in their study is similar to the mouse used in this study, however, the difference between the mouse model in the Perfitt et al.^58^ paper and ours is the newer MCK-*Fxn*^flox-flox^ mouse contains two alleles with a floxed exon 2 whereas ours contains a single allele with a floxed exon 2. Echocardiography parameters show worse ejection fraction as well as greater end diastolic volume and diameter in male KO compared to female KO mice. They also show slightly worse fibrosis in males compared to females. Further studies with these mouse models would be warranted to try to determine the reason for this sexual dimorphism, and specifically to see if there are similar differences in hormone levels, as we see in our study.

Our data suggest that the decline in circulating testosterone in *Fxn*-cKO mice negatively impacted cardiac function in males, while females were protected by the normal amount of estrogen. There is little to no information on sex hormone levels in FA patients. One report indicates the presence of significant erectile dysfunction in male FA patients, but the levels of testosterone were not measured in this study^59^. However, decreased levels of testosterone were reported in other types of ataxias such as Spinocerebellar Ataxia Type 2 (SCA2). Male patients with SCA2 showed a 35% reduction in testosterone level as compared to healthy male individuals (from 13.63 to 8.86 nmol/l), and this decrease in testosterone levels was associated with disease duration and age of ataxia onset^60^. In addition, there was a 38% decrease in circulating levels of the follicle-stimulating hormone (FSH, from 6.11 ± 4.13 in healthy males to 3.82 ± 3.06 mIU/ml in individuals with SCA2) and ~16% decrease in luteinizing hormone (LH) levels (from 4.36 ± 1.92 in healthy males to 3.65 ± 2.86 mIU/ml in SCA2 affected males).

We only found two studies where the careful analysis of cardiac parameters in patients with FA suggested a sex-specific difference between males and females^6,17^. One study which analyzed cardiac parameters in a small cohort of 80 FA patients based in the UK, revealed that the severity of the cardiac disease was associated with the increase in length of the shortest allele repeat (GAA1)^6^. Regression analysis revealed that a decline in left ventricular (LV) ejection fraction correlated with duration of the disease monitored for the duration of 20 years and was more pronounced in males compared to females (p = 0.002). No other cardiac parameters showed a significant difference between males and females except of near significant 16% increase (P = 0.054) in LV posterior wall thickness at diastole (LVPWd) in male FA patients as compared to females.

The second study was a cross-sectional study, where cardiac function was examined in 216 individuals with FA which included 68 children and 148 adults with an average GAA repeat size between 600 and 900^17^. The average age of children included in the study was 14 years old, and the average age for adults was 35. A significant increase in relative wall thickness (RWT) of the LV in both children and adults with FA was a common feature of the LV abnormality in the absence of the reduction in LV ejection fraction (LVEF). In adults with a normal LVEF, all LV variables other than RWT were larger in males independent of body surface area. When sex and body surface area were included in consideration, the length of GAA repeats inversely correlated with LV end-diastolic diameter, LV end-diastolic external diameter, LV end-diastolic length and LV end-diastolic volume (p < 0.01 for all), but not with LV mass. Several parameters of cardiac function including septal wall thickness, left ventricular mass, and LV end-diastolic length were higher in males with FA as compared to females even after adjusting for age and body surface area^17^.

Altogether, these data suggest that in addition to the length of GAA repeat, cardiac outcomes in FA were dependent on sex, age, and the duration of the disease with more severe cardiac parameters shown in males with FA relative to females. More importantly, we found that gene expression of critical excitation-contraction coupling proteins including L-type calcium channels, RyR2, SERCA2, PLN, and CaMKIIδ was decreased significantly more in *Fxn*-cKO males while expression of NCX1 was decreased to a similar degree in both males and females. In contrast to findings in “classical dilated heart failure” where expression of NCX1 and CaMKIIδ are typically upregulated, heart failure in FA was associated with the decreased expression in NCX1 and CaMKIIδ in both males and females which indicates that classical HF treatment regime may not work for cardiomyopathy in FA, and more detailed investigation on this topic is required.

## Conclusion

*Fxn-*cKO mouse model of FA recapitulates the cardiomyopathy with the reduced ejection fraction which is observed in FA patients at the late stage of disease and longer GAA repeat expansion. Similar to clinical findings of cardiac measures in patients with FA, a significant sexual dimorphism was present in *Fxn-*cKO mice as revealed by a premature death, more severe cardiac dysfunction and kidney failure in *Fxn-*cKO males as compared to KO females. The severity of cardiac and kidney dysfunction correlated with the lower levels of circulating testosterone and expression levels of mitochondrial proteins involved in cholesterol transport.

Due to the discovery of the differential expression of MCK and frataxin in *Fxn-*cKO males and females, it is critical to examine sex differences when conducting drug testing in this mouse model of FA. *Fxn*-cKO males developed more severe cardiomyopathy and died sooner, which we hypothesize is due to severe decline in testosterone production and kidneys failure which was not observed in KO females. Further studies need to be conducted with testosterone and estrogen supplementation to determine the specific role of these hormones in the development of cardiomyopathy in this mouse model.

## Methods

### Animal model and care

All animal handling and laboratory procedures were in accordance with the approved protocols of the Institutional Animal Care and Use Committee of the University of California, Davis conforming to the Guide for the Care and Use of Laboratory Animals published by the US National Institute of Health (8th Edition, 2011).

For all studies, phenotypically normal parental strains were obtained from The Jackson Laboratory and bred in our animal facility. Stock #028520 (homozygous floxed frataxin exon 2) and stock #029100 (heterozygous frataxin global knockout and homozygous MCK-Cre) were bred to obtain the experimental *Fxn*^*flox/null*^*::MCK-Cre* mice animals (floxed frataxin exon 2 and global knockout on respective homologous chromosomes, hemizygous for *MCK-Cre* similar to the stock # 029720), referred to here as *Fxn*-cKO mice. We used littermates with floxed exon 2 and wild-type frataxin on respective homologous chromosomes, hemizygous for *MCK-Cre* as control (CTRL) animals in this study. Control animals are phenotypically normal with no known or expected effects on lifespan or function. Animals were genotyped according to the Jackson Laboratory’s protocol. Mice were housed in a temperature- and humidity-controlled animal facility, with a 12-h light-dark cycle and free access to deionized water and standard grain-based rodent chow (Teklad 2018, Inotivco). Animals were monitored daily in survival studies. Mouse weights were evaluated weekly. Euthanasia was performed with pentobarbital overdose, with exsanguination under deep anesthesia provided by the overdose. We have complied with all relevant ethical regulations for animal use.

### Echocardiography

Cardiac function was examined using 2D echocardiography using a VisualSonics Vevo 2100 echocardiography machine (VisualSonics, Toronto, ON, Canada) and a MS 550D probe (22–55 MHz) under anesthesia with 1–1.5% isoflurane in a 100% O_2_ atmosphere gas chamber. We used the parasternal short- and long-axis views to obtain 2-dimensional and M-mode images^61,62^. M-mode imaging was done in conscious animals to exclude the effects of anesthesia on cardiac function. Left ventricular dimensions EDD, end diastolic dimension; ESD, end systolic dimension; PWT, posterior wall thickness; were obtained from 2D guided M-mode images. Indices of contractility such as left ventricular (LV) fractional shortening (FS), ejection fraction (EF), stroke volume, and cardiac output were calculated from the M-mode images. Left ventricular (LV) mass was calculated using M-mode data using the following equation: LV mass =1.05 ([LVIDD + LVPWT + IVSD]^**3**^ − [LVIDD]^**3**^), where LVIDD is LV internal diameter at diastole, LVPWT is LV posterior wall thickness at diastole and IVSD is intraventricular septum thickness at diastole^61^.

### Serum biochemical profiling

Serum biochemistry panel was performed by UC Davis Comparative Pathology Laboratory using a Roche Integra 400 Plus. This panel includes measurements of amylase, blood urea nitrogen (BUN), calcium, chloride, creatinine, sodium, phosphorus, bilirubin, alkaline phosphatase (ALP) and creatine kinase.

### Circulating hormones and vitamin D detection

Serum Testosterone and estradiol concentration was measured by R&D Systems ELISA kits KGE010 and KGE014, respectively. Serum levels of 25(OH)Vitamin D were detected with Cayman Chemicals ELISA kit.

### Mitochondrial assays

Mitochondrial Complex I and II activity assay and ATP assay were performed according to manufacturer’s instructions from Abcam (ab109721, ab109908, and ab83355, respectively). Aconitase activity was determined with Abcam assay (ab109712).

### Quantitative real-time PCR

RNA was extracted from crushed, frozen hearts, using RNeasy Fibrous Tissue Kit (Qiagen, Hilden, Germany) or crushed, frozen kidney using RNeasy Plus Universal Kit (Qiagen, Hilden, Germany) according to manufacturer’s instructions. cDNA was created using iScript cDNA Synthesis kit (BioRad Laboratories, Hercules, CA, USA). Quantitative RT-PCR was carried out using PowerUp Sybr Green (ThermoFisher Scientific, Waltham, MA, USA) on a QuantStudio 7 Pro Real-Time PCR System (ThermoFisher, Waltham, MA, USA). The data was analyzed with a delta-delta CT calculation using ribosomal protein 18S as a reference gene for heart and β-actin for kidney. Primer sequences can be found in the Supplementary Table 1.

### Protein expression

Protein was extracted using Cell Lytic lysis buffer (Sigma Aldrich, St. Louis, MO, USA) containing PhosSTOP phosphatase inhibitor and cOmplete protease inhibitor (Roche, Basel, Switzerland). Protein concentration was determined by Pierce BCA Protein Assay (ThermoFisher Scientific, Waltham, MA, USA). Protein separation, detection, and analysis was done using Jess-Chemiluminescent & Fluorescent Jesstern Blot system by Protein Simple (Biotechne, Minneapolis, MN, USA). Rabbit monoclonal anti-frataxin antibody (1:100 dilution) (gifted by Dr. Franco Taroni), and anti-αTubulin (1:200 dilution) (Sigma Aldrich, St. Louis, MO, USA) were used as primary antibodies, and anti-mouse NIR and anti-rabbit HRP were used as secondary antibodies (Protein Simple). All lysates and solutions were plated according to manufacturer’s instructions.

### Statistical analysis and reproducibility

Data were analyzed using two-way ANOVA unless otherwise noted using Graphpad Prism Version 10.0 (Graphpad Software Inc. San Diego, CA) using p < 0.05 as statistically significant. Data are presented as mean ± standard error of the mean (SEM). All qRT-PCRs and Jesstern blots were run twice to ensure reproducibility. Serum markers were run on two separate cohorts of animals to ensure reproducibility. n = 4–10 animals per group depending on the experiment.

### Reporting summary

Further information on research design is available in the Nature Portfolio Reporting Summary linked to this article.

## Supplementary information

Supplemental Material

Description of Additional Supplementary Materials

Supplementary Data 1

Supplementary Data 2

Reporting Summary

## Data Availability

The datasets generated and analyzed during the current study including the sequences of the oligonucleotide primers are available in the Supplementary Materials. All source data underlying the graphs and charts presented in the main and Supplementary Figs. are available in Supplementary Data 1 and 2.
