# Supplementary material for: Sexual dimorphism in a mouse model of Friedreich’s ataxia with severe cardiomyopathy
Source: Commun Biol. 2024 Oct 3;7:1250. doi: 10.1038/s42003-024-06962-4 (PMC11449905; doi:10.1038/s42003-024-06962-4)
Supplement: Supplementary file 1 — Supplemental Material [file 42003_2024_6962_MOESM1_ESM.pdf]

Supplementary Materials

Sexual dimorphism in a mouse model of Friedreich's ataxia with severe cardiomyopathy

Running title: *Salinas et al.; Sexual dimorphism in Friedreich's ataxia*

Lili Salinas[1], Claire B. Montgomery[1], Francisco Figueroa[1], Phung N. Thai[2,3], Nipavan Chiamvimonvat[2,3], Gino Cortopassi[1], Elena N. Dedkova[1,4]

[1]Department of Molecular Biosciences, University of California, Davis, CA, USA;
[2]Department of Internal Medicine, University of California, Davis, CA, USA;
[3]Department of Veterans Affairs, Northern California Health Care System, Mather, CA, USA;
[4]Department of Basic Sciences, California Northstate University, Elk Grove, CA, USA

Journal Subject Terms: Animal Models of Human Disease; cardiomyopathy; Friedreich's ataxia, sexual dimorphism, frataxin, muscle creatine kinase.

The following is included in the supplementary materials.

Supplementary Figure S1
Supplementary Figure S2
Supplementary Figure S3
Supplementary Table 1

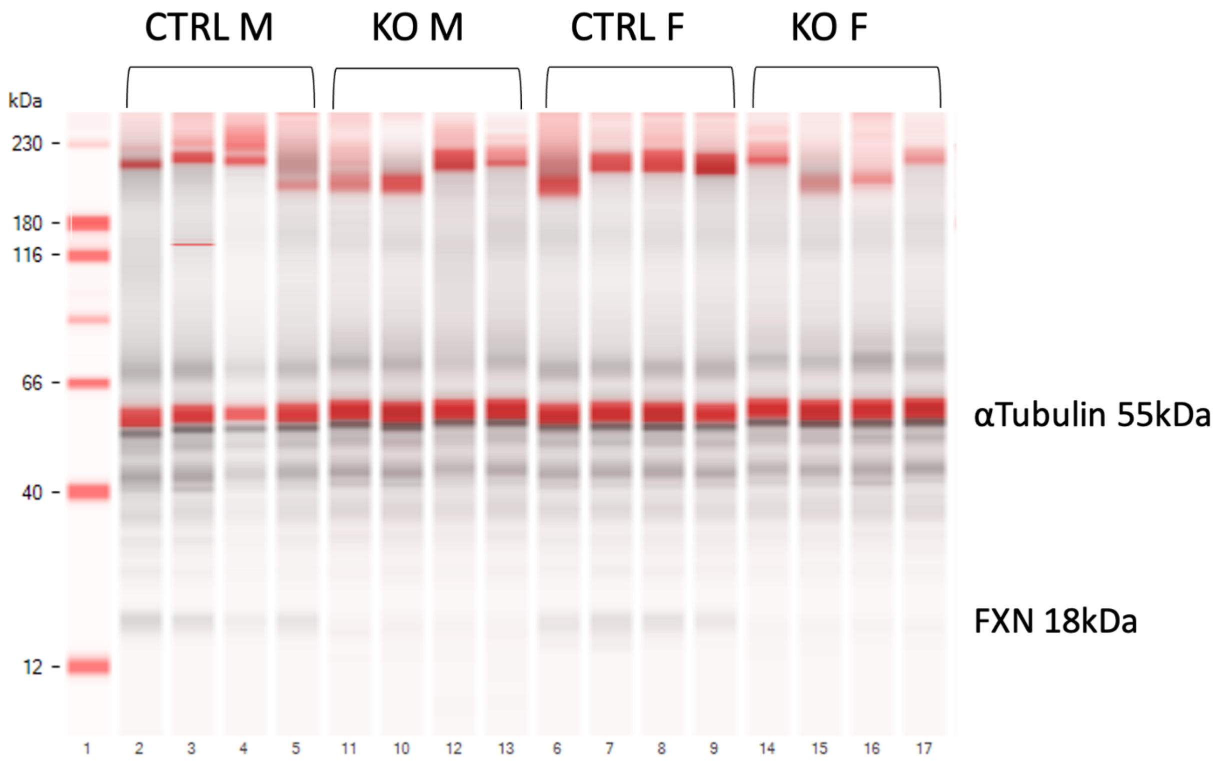

Supplemental Fig. 1 Representative uncropped Jesstern blot of frataxin protein expression in control and *Fxn*-cKO male (left) and female (right) hearts. αTubulin was used for frataxin expression normalization. n=4 animals per group.

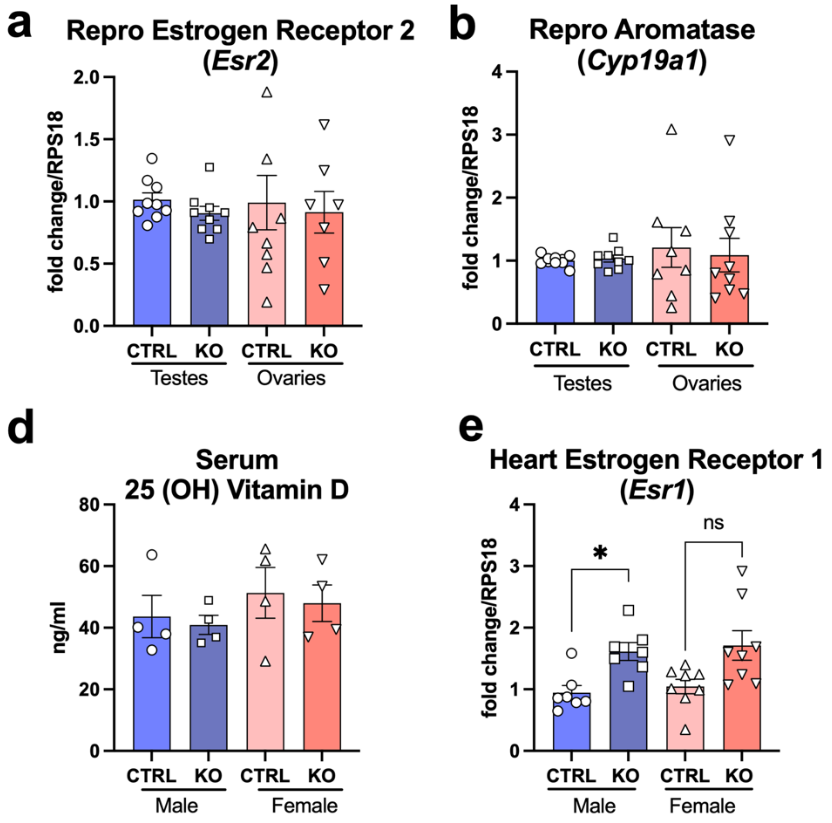

Supplemental Fig. 2 a Gene expression of estrogen receptor 2 (*Esr2*) in testes and ovaries of CTRL males (n=9), *Fxn*-cKO males (n=9), CTRL females (n=9) and *Fxn*-cKO females (n=9). **b** Gene expression of aromatase (*Cyp19a1*) in testes and ovaries of CTRL males (n=9), KO males (n=9), CTRL female (n=9), and KO females (n=9). **c** Gene expression of glutathione S-transferase pi 1 (*Gstp1*) in testes and ovaries of control and *Fxn*-cKO mice. **d** 25 (OH) Vitamin D measurements in serum of control and Fxn-cKO animals, n=4 per group. **e** Gene expression of estrogen receptor 1 (ESR1) in the heart of CTRL males (n=7), *Fxn*-cKO males (n=7), CTRL females (n=8) and *Fxn*-cKO females (n=8). Statistical analysis was done by two-way ANOVA with *p<0.05 and **p<0.01. NS- non-significant.

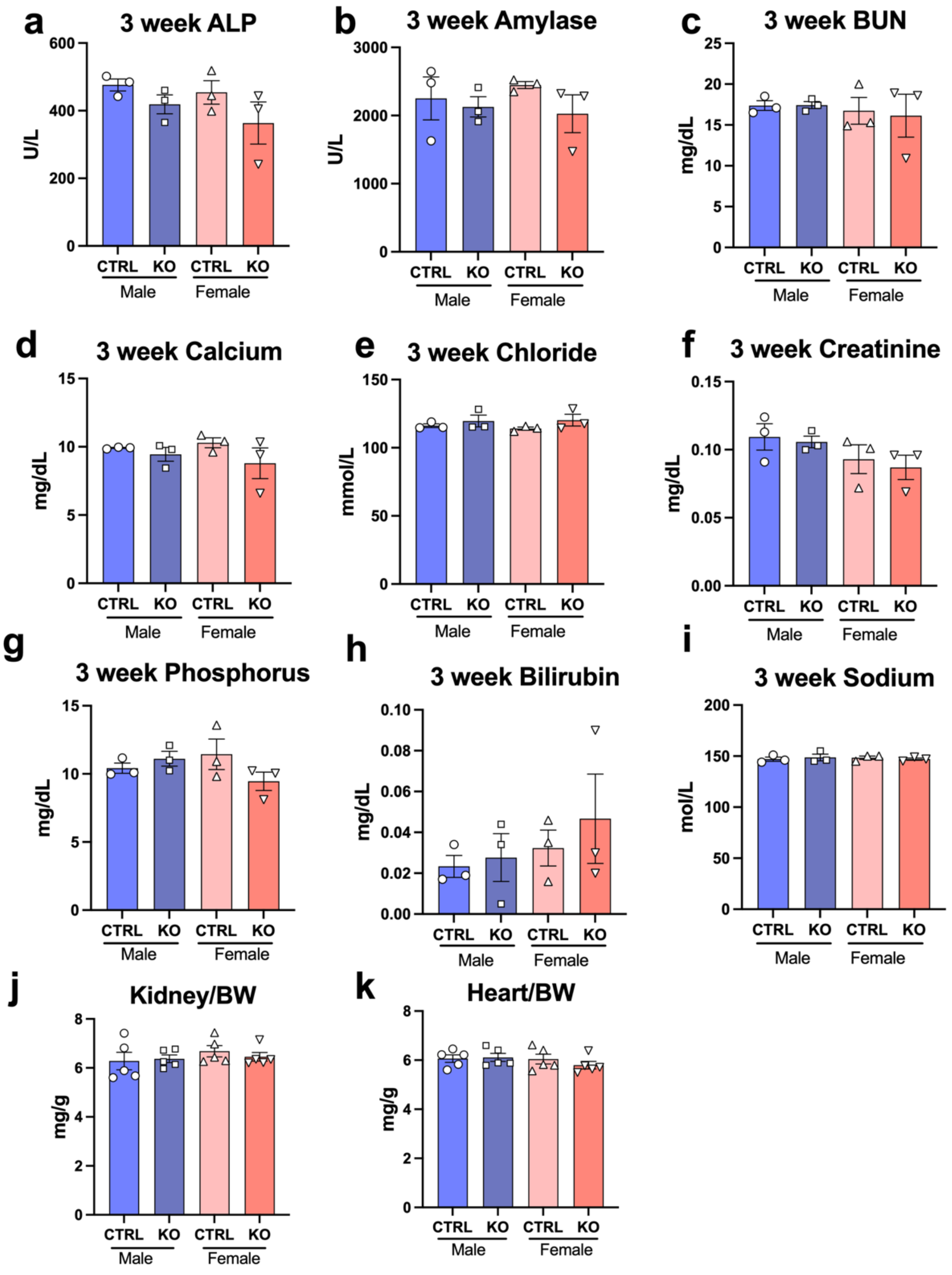

Supplemental Fig. 3 Summary of serological and morphological analysis in 3-week-old control and *Fxn*-cKO mice. a Serum ALP levels in 3-week-old control and *Fxn*-cKO mice. **b** Serum amylase levels. **c** Serum blood urea nitrogen (BUN) levels. **d** Serum calcium levels. **e** Serum chloride levels. **f** Serum creatinine levels. **g** Serum phosphorus levels. **h** Serum bilirubin levels. **i** Serum sodium levels. **j** CTRL and *Fxn*-cKO mouse kidney weight normalized to body weight in 3-week-old animals. **k** CTRL and KO mouse heart weight normalized to body weight in 3-week-old animals. 3-5 animals per group were included in measurements as shown by symbols.

Supplemental Table 1. Primer Sequences

Gene name	Forward (5'-3')	Reverse (5'-3')
Fxn	GATCAACAAGCACCCAAA	AGGCCAATGAAGGCGAGTCCA
Nppb	GGCAAGTTTCTCCAAGA	ACTCTGCCTGGGTCTCTTTC
Nos3	GGCTGGTTTAGGGCTGTG	CTGAGGGTGTCGTAGGTGATG
Cacna1c	TCTGCTTCCGAAGATGA	AGGTGACATGGTTGCCGAAC
Cacna1d	TGTGGCCATCCGAACCATTG	CCCTGCACTCCTCAGGGTTA
Lgals3	TGTTGAGAGATACCCATCGC	GTACCCATGTGCACAGACAC
Postn	GCGAAGAAAGGGAGCTTCTG	CCCTGTGTGGTCTTCAT
Cyp11a1	TCGGCTTTTCCTTTGAGTCCA	CTGCCAGCATCTCGGTAATGT
Cyp19a1	CCTGACGAAAGAGAACGTGAA	GCTTCGACCTCTGGATACTC
Fdx1	ACAAACTTGGCAGCCCAAC	GAAAATTGGCGACTCTCTGC
Nqo1	ACAGGTGAGCTGAAGGACTC	CCAAACCACTGCAATGGGAA
Gstp1	GACCTGCTGCTGATCCACCA	AGTGCTGGGAAAACGGGGAC
Ar	AGGAGAAAACTCCAATGCTGG	TCTGGTTGGTTGTTGTCATGT
Esr1	TCTGCCAAGGAGACTCGCTA	GTGCATTGGTTTGTAGCTGGA
Esr2	TCGTTCTGGACAGGGATGAG	ATCATGGCCTTCACACACAG
Tspo	TGGGAGGTTTCACAGAGGAC	CAAGCAGAAGATCGGCCAAG
Star	GGCATACTCAACAACCAGGAA	CCTTGACATTTGGGTTCCACT
Ryr2	GGCCACATGGACGATGGTTT	TAGCCGATGAGGTCCTGACAA
Atp2a2	CTTTGCCGCTCATTTTCCAG	AGGCTGCACACACTCTTTAC
Camk2d	ATATCCTCCTGGTGGGCTAC	AATCGTAGGCTCCAGCTTTG
Ncx1	ACTTCCTTGTGCATCTTAGCA	GTCACGGGTTCCTCAAATGT
Pln	CTCGCTCGGCTATCAGGAGA	TTGGCATGTTGCAGGTCTGG
Rps18	ATGCAGAACCCACGACAGTA	TTCTTCAGCCTCTCCAGGTC
Actb	TTACTGCTCTGGCTCCTAGC	CCTGCTTGCTGATCCACATC
