# Supplementary material for: Sexual dimorphism in a mouse model of Friedreich’s ataxia with severe cardiomyopathy
Source: Commun Biol. 2024 Oct 3;7:1250. doi: 10.1038/s42003-024-06962-4 (PMC11449905; doi:10.1038/s42003-024-06962-4)
Supplement: Supplementary file 2 — Description of Additional Supplementary Materials [file 42003_2024_6962_MOESM2_ESM.pdf]

Description of Additional Supplementary Files

File name: Supplementary Data 1

Description: contains all data sets shown in the Main body of the manuscript.

File name: Supplementary Data 2

Description: contains all data sets shown in the Supplementary Materials.
